# Magnetite Alters the Metabolic Interaction between Methanogens and Sulfate-Reducing Bacteria

**DOI:** 10.1021/acs.est.3c05948

**Published:** 2023-10-20

**Authors:** Ginevra Giangeri, Panagiotis Tsapekos, Maria Gaspari, Parisa Ghofrani-Isfahani, Marie Karen Tracy Hong Lin, Laura Treu, Panagiotis Kougias, Stefano Campanaro, Irini Angelidaki

**Affiliations:** † Department of Chemical and Biochemical Engineering, Technical University of Denmark, DK-2800 Kgs. Lyngby, Denmark; ‡ Department of Hydraulics, Soil Science and Agricultural Engineering, Faculty of Agriculture, Aristotle University of Thessaloniki, GR-54124 Thessaloniki, Greece; § National Centre for Nano Fabrication and Characterization, Technical University of Denmark, Kgs, DK-2800 Lyngby, Denmark; ∥ Department of Biology, University of Padova, Via U. Bassi 58/b, 35121 Padua, Italy; ⊥ Hellenic Agricultural Organization Dimitra, Soil and Water Resources Institute, Thermi, GR-54124 Thessaloniki, Greece

**Keywords:** anaerobic digestion, direct interspecies electron transfer, conductive materials, genome-centric metagenomics, sulfate-reducing bacteria

## Abstract

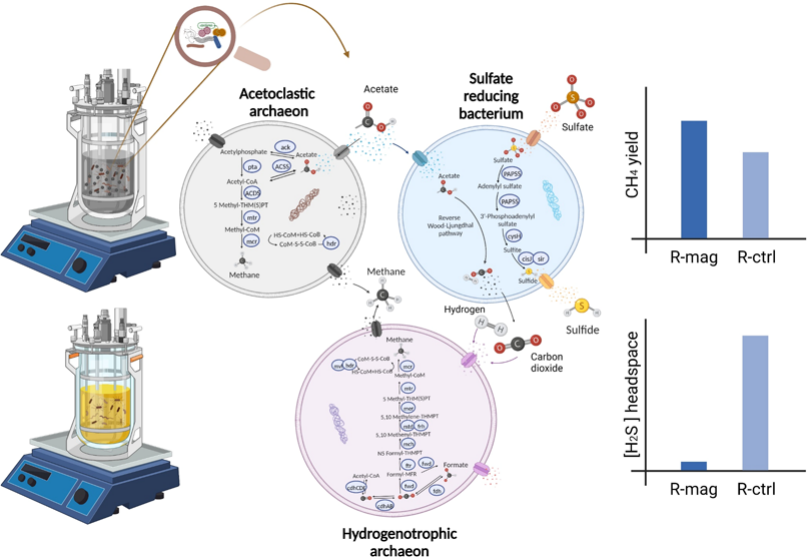

It is known that the presence of sulfate decreases the methane yield in the anaerobic digestion systems. Sulfate-reducing bacteria can convert sulfate to hydrogen sulfide competing with methanogens for substrates such as H_2_ and acetate. The present work aims to elucidate the microbial interactions in biogas production and assess the effectiveness of electron-conductive materials in restoring methane production after exposure to high sulfate concentrations. The addition of magnetite led to a higher methane content in the biogas and a sharp decrease in the level of hydrogen sulfide, indicating its beneficial effects. Furthermore, the rate of volatile fatty acid consumption increased, especially for butyrate, propionate, and acetate. Genome-centric metagenomics was performed to explore the main microbial interactions. The interaction between methanogens and sulfate-reducing bacteria was found to be both competitive and cooperative, depending on the methanogenic class. Microbial species assigned to the *Methanosarcina* genus increased in relative abundance after magnetite addition together with the butyrate oxidizing syntrophic partners, in particular belonging to the *Syntrophomonas* genus. Additionally, *Ruminococcus* sp. DTU98 and other species assigned to the Chloroflexi phylum were positively correlated to the presence of sulfate-reducing bacteria, suggesting DIET-based interactions. In conclusion, this study provides new insights into the application of magnetite to enhance the anaerobic digestion performance by removing hydrogen sulfide, fostering DIET-based syntrophic microbial interactions, and unraveling the intricate interplay of competitive and cooperative interactions between methanogens and sulfate-reducing bacteria, influenced by the specific methanogenic group.

## Abstract 2

This study shows how magnetite can improve methane production and remove hydrogen sulfide in anaerobic digestion systems, shedding light on the interaction between sulfate-reducing bacteria, methanogens, and their syntrophic partners. These findings have environmental implications for the use of conductive materials in anaerobic digestion and for waste recycling.

## 1 Introduction

Anaerobic digestion (AD) is a complex process involving diverse microbiota that degrades a heterogeneous mixture of compounds producing methane (CH_4_). In brief, the hydrolysis of complex organic matter leads to the formation of simpler molecules that undergo a primary fermentation to obtain acetate, carbon dioxide (CO_2_), hydrogen (H_2_), and formate, which are then secondarily used to obtain CH_4_.^1^ The fine balance standing at the root of methanogenesis can be disturbed either by inhibition, such as high ammonia or volatile fatty acids concentrations, or by competition, such as the one between methanogens and sulfate-reducing bacteria—SRB.^2,3^ In anaerobic bioreactors, the presence of sulfur compounds such as protein, sulfate, thiosulfate, and sulfite leads to the formation of highly toxic and corrosive hydrogen sulfide (H_2_S), but there is still no definitive answer to the removal of this compound.^4^ Sulfate (SO_4_^2–^) is reduced to H_2_S by SRB, which consume the same substrates used by methanogens, including acetate, and electron donors, including formate and H_2_, indeed creating a competitive behavior and ultimately limiting biomethane production.^5^ Methanogenesis based on indirect interspecies electron transfer (IIET)—comprising interspecies hydrogen transfer and/or interspecies formate transfer—is a rate-limiting step, since the highly energetic electron donors, such as formate and H_2_, are produced exclusively when their concentration is kept low. Additionally, SRB overcompete methanogens since sulfate reduction is energetically more favorable compared to CO_2_ reduction.^6^ An alternative to IIET-based methanogenesis is a syntrophic mechanism based on direct interspecies electron transfer (DIET) where exoelectrogenic bacteria can directly provide electrons to electrotrophic archaea.^7^

It has been previously assessed that in the methanogenic DIET-based process, electrically conductive pili (e-pili) associated with multiheme c-type cytochromes (MHCs) play a fundamental role.^8,9^ These membrane-bound conductive structures are extensively involved in the extracellular transfer of electrons at the biotic–abiotic interfaces both in bacteria and archaea.^8,10,11^ However, it was recently revealed that the identification of DIET-capable microorganisms is not restricted to the presence of e-pili and MHCs.^12^ In fact, specific electron conductive structures external to the microorganisms, known as conductive materials (CMs), have the potential to enhance the electroactive properties of anaerobic biofilms.^13,14^ Examples of such conductive materials are humic acids, metallic ion content in the membrane, and biofilm polymers. However, the molecular mechanism of electron transfer in these systems is not yet fully understood. Examples are provided by humic compounds that undergo multiple reduction and oxidation steps, thereby transferring electrons from one microbial cell to another, while magnetite can form wires connecting the cells and allowing the electron flow, without the requirement of redox reactions.^12,15^ A classification can be proposed for carbon-based CMs such as biochar, granular activated carbon (GAC), carbon nanotubes (CNTs), and non-carbon-based CMs such as magnetite (Fe_3_O_4_) and stainless steel.^16^ As a non-carbon-based iron oxide, magnetite is a ferrimagnetic mineral usually found in the form of Fe_3_O_4_ that improves electron transfer ability by replacing the OmcS proteins distributed in the e-pili.^17^ Additionally, the position of the iron atom in the magnetite molecule can provide conductive properties exploitable for electron transfer between syntrophs; this can result in an increased degradation rate of organic matter during AD.^18^ There is overwhelming evidence that magnetite addition in methanogenic DIET-based systems has multiple positive effects, including, for instance, an increase in the CH_4_ production rate, a reduction of the lag phase, and more efficient removal of many toxic intermediate compounds (e.g., benzoate, phenanthrene).^19,20^ However, to our knowledge, the effect of magnetite on the competition between SRB and methanogens is still to be clearly elucidated as well as the impact of this compound on the H_2_S removal and the overall dynamics occurring in complex microbiota.

The central aim of this work was to elucidate how the introduction of magnetite as a conductive material would promote DIET mechanisms, thereby influencing the intricate interactions between methanogens and SRB. Consequently, the addition of magnetite was expected to have significant effects on the competitive dynamics between methanogens and SRB, as well as on the removal of H_2_S, ultimately impacting the overall composition and behavior of the microbiota during anaerobic digestion processes. For disclosing these interactions, two continuously stirred tank reactors (CSTRs) were monitored during long-term operation. At steady-state conditions, sulfate (SO_4_^2–^) shocks were applied to stress the methanogenic population and thereby decrease the methane yield. Subsequently, magnetite was added as a conductive material to potentially promote DIET. Time series metagenomic analyses were applied to appreciate the microbiome evolution. A better comprehension of the interactions between SRB and methanogens is provided in the context of AD and the effect of magnetite on the microbial community is revealed.

## 2 Materials and Methods

### 2.1 Inoculum, Feedstock, and Experimental Setup

The inoculum used in this study was obtained from the Hashøj Biogas plant (Denmark), stocked in 5 L plastic tanks, and rapidly transferred to the laboratory. It was sieved with a 5 mm sieve to remove large fibers and avoid blocking of tubes and then sparged with gaseous N_2_ for 30 min to ensure an anaerobic environment. Source-separated organic waste was collected in the form of municipal biopulp from HCS A/S Transport & Spedition (Glostrup, Denmark) and used as feedstock. Biopulp was sieved with a 5 mm diameter mesh, diluted with distilled water to a final concentration of 56 gVS/L, then stored at −20 °C, and thawed at 4 °C before use. The characteristics of the inoculum and the feedstock are reported in Table S1 (Data S1).

Two identical lab-scale CSTRs (R-ctrl and R-mag) of 1.8 L working volume (2.3 L total volume) were set up. Both CSTRs consisted of the reactor vessel equipped with a magnetic stirrer, an influent bottle with a stirrer to ensure substrate homogeneity, a peristaltic pump for feeding, an effluent bottle, an electrical heating jacket, and a water-displacement gas meter. The reactors were operated under mesophilic (37 ± 1 °C) conditions to maintain the same working temperature as that operated in the biogas plant. The hydraulic retention time (HRT) was set at 23 days by a daily supply of 70 mL of diluted biopulp, leading to a constant organic loading rate (OLR) of 2.30 gVS/L-reactor.day. The experimental period was divided into four phases, and the reactors were maintained under the same conditions in the first three phases, while in the last phase, magnetite was added only in one of the two CSTRs: P1 (days 0–44); P2 (days 45–68); P3 (days 69–109), and P4 (days 110–197). The reactors were running at a steady state (less than 10% methane production variation for at least ten consecutive days) throughout phase P1. At the beginning of P2, Na_2_SO_4_ (sodium sulfate suitable for HPLC, LiChropur, 99.0–101.0%, Sigma-Aldrich) was added as a single pulse to reach 0.6 g SO_4_^2–^/L in both CSTRs. Subsequently, the CSTRs were fed with Na_2_SO_4_-rich biopulp to maintain the sulfate content at the desired value. The second shock occurred at P3 when the Na_2_SO_4_ level was increased to 1.2 g of SO_4_^2–^/L in both CSTRs and feedstocks. In P4, the content of SO_4_^2–^ was kept constant and magnetite (Iron(II, III) oxide powder, <5 μm, 95%, Sigma-Aldrich) was added progressively (day 110 to 3.3 g/L, day 115 to 6.6 g/L, and day 120 to 10 g/L) only in R-mag for 12 days to reach a final concentration of 10 g/L. During this period, both reactors were manually fed from day 110 to day 122 and on days 110, 112, 114, 120, and 122, 3.6 g of magnetite were added in R-mag. In order to add SO_4_^2–^ and magnetite, the feeding through the pumps was stopped, and the reactors were manually fed using a syringe containing diluted biopulp mixed with the compound of interest. The manual feeding was performed with 8 h gap within the same day and 16 h gap between one day and the other. The use of the syringe allowed to maintain anaerobic conditions while manually feeding.

### 2.2 DNA Extraction, Shotgun Sequencing, and Genome-Centric Metagenomics

Samples of 15 mL were collected at the end of each phase for DNA extraction, named R-mag P1, R-ctrl P1, R-mag P3, R-ctrl P3, R-mag P4, and R-ctrl P4. Genomic DNA was isolated and purified using the DNeasy PowerSoil (QIAGEN 181 GmbH, Hilden, Germany) following the manufacturer’s protocols with minor modifications.^21^ Genomic DNA quality and quantity were determined using a Multiskan Sky Microplate Spectrophotometer (operated with Thermo Scientific SkanItTM Software 5.0, Thermo Fisher Scientific) and a Qubit fluorometer (Life Technologies, Carlsbad, CA). Illumina libraries were prepared with the Nextera DNA Flex Library Prep kit (Illumina, Inc., San Diego CA) and sequenced on the Illumina NovaSeq platform, producing 150 bp paired-end reads at the NGS sequencing facility of the Biology Department (University of Padova, Italy).

Reads quality was assessed using FastQC Software (v0.11.9). Reads in FASTQ format were quality-filtered (leading:20, trailing:20, slidingwindow:4:20, minlen:100) and adapters were removed using Trimmomatic software (v0.39).^22^ Assembly, SAM files, sorting, and indexing of BAM files were performed following Yan and colleagues.^22^ Four different tools were included in the binning approach: Concoct (v1.1.0), Maxbin2 (v2.2.7), MetaBAT (v1:12.1), and MetaBAT2 (v2:12.1).^23−26^ DASTool (v1.1.5) and Binning_refiner (v1.4.2) were used to integrate the results of the binning algorithms to calculate an optimized, nonredundant set of bins.^27,28^ CheckM (v1.1.6) was used to assess the quality of MAGs (lineage_wf) and their relative abundance in each sample.^29^ Differences in relative abundance were visualized and analyzed using Multiple Experiment Viewer (MeV) (v4.9.0) and clustering was performed using Pearson correlation and average linkage.^30^ Taxonomy was assigned to the MAGs using GTDB-Tk (v2.1.0) and was subsequently converted to NCBI taxonomy.^31^ Gene prediction was performed with Prodigal (v.2.6.3) (REF) Functional annotation was performed with both EggNOG (v.1.0.3) (REF) and Anvi’o.^32^ KEGG functional analysis was refined using GhostKOALA.^33^ Metabolic pathways enrichment and enzymes associated with DIET were identified by performing comparisons with enrichM “annotate” and “enrichment” software (v0.6.4).^34^ Sequence data reported in this study have been submitted to the National Center for Biotechnology Information under BioProject PRJNA911044.

### 2.3 SEM Analysis and Qualitative Sulfur Detection

The wet catalytic oxidation reaction between magnetite and dissolved H_2_S was assessed by performing an independent experiment in sterile conditions.^35^ Basal anaerobic (BA) medium was prepared according to Angelidaki and Sanders,^36^ 50 mL was added to a 200 mL bottle and purged with N_2_ for 15 min before autoclaving. Magnetite and H_2_S were added to the bottles at two different S/Fe ratios: 1:9 to simulate the reactor’s state (R-mag, c[SO_4_^2–^] = 1.2 g/L, c[Fe_3_O_4_] = 10 g/L) and 1:18 to test the excess concentration of magnetite. In addition, one control bottle without magnetite was set up to assess the dissolution of H_2_S in the liquid phase. The volume of H_2_S added per bottle was kept constant (160 mL, at standard temperature and pressure), and the amount of magnetite was changed to examine the different conditions. After the gas addition, the pressure inside the bottles was around 1.6 bar (Table 1). Pressure was monitored in triplicate at different time intervals over 24 h (immediately after H_2_S addition, after 2 and 24 h) using HD2124.2 manometer (Delta OHM). At the end of the experiment, magnetite was collected from the bottle, placed on support for SEM analysis, and dried in a desiccator. SEM images were taken at the DTU Nanolab (National Centre for Nano Fabrication and Characterization) with an FEI Quanta FEG 200 microscope at 10 kV with a spot size of 3.5 and a working distance of about 10 mm. EDX analysis had the same parameters as the SEM analysis and was detected by Oxford Instruments 80 mm2 X-Max Silicon drift detector, MnKα resolution at 124 eV to evaluate the presence of sulfur compounds associated with the magnetite.

### 2.4 Analytical Methods

The American Public Health Association (APHA) standard methods were followed to measure total solids (TS), volatile solids (VS), and volatile suspended solids (VSS).^37^ Biogas composition was analyzed with a gas chromatograph (GC-TRACE 1310, Thermo Fisher Scientific) equipped with a thermal conductivity detector (TCD) and Thermo (P/N 26004–6030) Column (30 m length, 0.320 mm inner diameter, and film thickness 10 μm) with helium as carrier gas (detection limit 0.0001%). TVFA (propanol, butanol, hexanol, acetate, propionate, isobutyrate, butyrate, isovalerate, valerate, hexanoic acid) concentrations were measured using an Agilent 7890A gas chromatograph (Agilent Technologies, US) equipped with a flame ionization detector (FID) and SGE capillary column (30 m length, 0.53 mm inner diameter, film thickness 1.00 μm) with helium as the carrier gas. The injection volume was 1 μL, and every sample was analyzed in duplicate to ensure the precision and reproducibility of the measures (quantification limit, 1 mg/L). The injector temperature was kept at 150 °C and the detector temperature was at 220 °C. The initial temperature of the column oven was held at 45 °C for 3.5 min, increased to 210 °C at a ramping rate of 15 °C/min, and then held for 4 min at 210 °C. The concentration (obtained in mg/L) of each detected VFA was summed up to obtain the TVFA concentration. pH trend was monitored using FiveEasy Plus Benchtop FP20 (Mettler Toledo, CH). H_2_S accumulation in the headspace of the reactors was measured with Geotech BIOGAS 5000 portable gas monitor (detection limit 1 ppm, QED Environmental Systems Ltd., U.K.). In particular, 100 mL of biogas was collected from the headspace and diluted with 700 mL of N_2_ into a gasbag, and the mixed gases were passed into the instrument for measurement.

### 2.5 Statistical Analysis

Descriptive statistics were conducted for all variables and mean values, and standard deviations were calculated. Wilcoxon signed-rank test has been performed to compare the values between the two reactors, and the Mann–Whitney U test was performed to compare the data of each reactor in the different phases. MAGs were classified into subgroups according to their 2-fold change within and between the different samples to select the groups of MAGs to be compared (Data S4 and S5): (1) fold-change >2 in R-mag in P4, (2) fold-change <2 in R-mag in P4, (3) fold-change >2 in R-ctrl in P4, (4) fold-change <2 in R-ctrl in P4. Pathways and single enzymes enriched in selected groups of MAGs were identified by performing comparisons with enrichM “enrichment” software (v0.6.4–0) according to the statistical results obtained with the Mann–Whitney U test.^38^

## 3 Results and Discussion

### 3.1 AD Performance and Effect of the Sulfate Shocks

**Figure 1:**
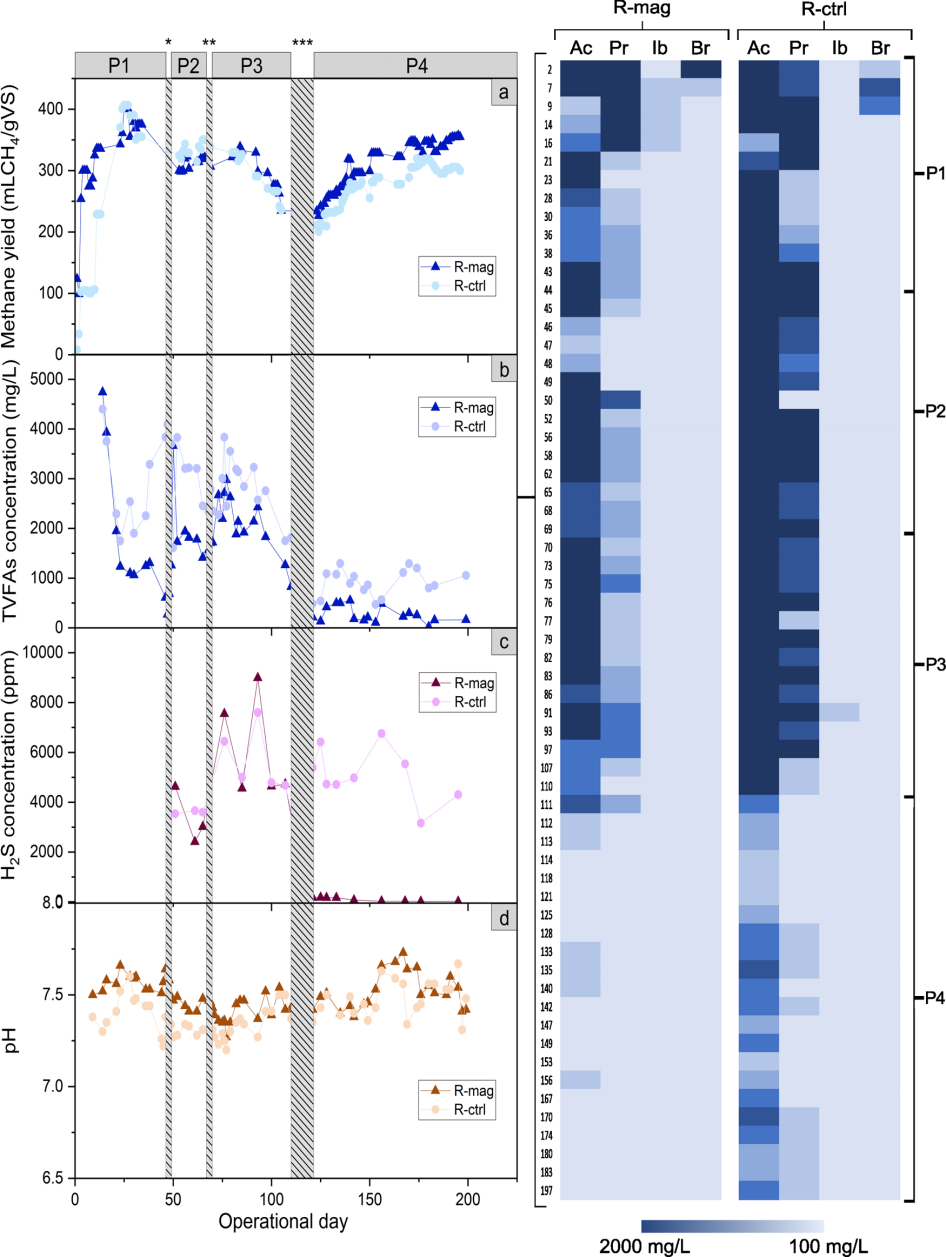
Overall CSTRs performance during the four phases (P1–P4). On the left part of the panel, four plots describe: (a) methane yield measurements, (b) TVFA trend, (c) H_2_S concentration after the first shock with 0.6 g/L SO_4_^2–^, and (d) pH drift. The (*) symbol marks the first SO_4_^2–^ shock at operational day 49, (**) the second shock to a final SO_4_^2–^ concentration of 1.2 g/L at day 66, and (***) points out the magnetite addition to a final concentration of 10 g/L, continuously applied for 12 days, from day 110 to 122. Data during the days of SO_4_^2–^ (day 44 and day 69) and magnetite (days 110–122) additions are not shown. Data are masked (gray dotted lines) to avoid discrepancies that might have arisen due to the different feeding procedure (e.g., manual compared to the automatic feeding through the pumps). On the right part, a heatmap representing the variation of the four main VFA identified: Ac (acetate), Pr (propionate), Ib (Isobutyrate), Br (butyrate). On the far right, squared brackets divide the heatmap into the four phases analyzed.

During P1, after the initial adaptation phase that lasted 13 days, both reactors performed similarly from day 14 to day 45 (*p*-value < 0.05), achieving a steady state with average methane yields of 375 ± 19 mL CH_4_/gVS and 381 ± 24 mL CH_4_/gVS for R-mag and R-ctrl, respectively (Figure 1a). After the first Na_2_SO_4_ supplementation at 0.6 g of SO_4_^2–^/L, the methane yield decreased by 15 ± 2% in both reactors. When the reactors reached a new steady state, Na_2_SO_4_ was added to reach a final concentration of 1.2 g SO_4_^2–^/L. The methane yield further decreased in both reactors by 20 ± 2% compared to P1. The raised SO_4_^2–^ concentration boosted SRB metabolism, resulting in more disturbed conditions for the methanogens and increased process instability. Indeed, SRB consume the same substrates as methanogens and a potential enrichment in their activity can hamper the methanogenesis.^39^ Nevertheless, the average percentage of CH_4_ in the biogas did not experience major fluctuations, maintaining average values around 62% ± 1% in both reactors. After the Na_2_SO_4_ addition, H_2_S started increasing from 500 ± 200 ppm to an average of 5000 ± 200 ppm (Figure 1c). The sudden rise suggested that SRB were substantially favored in their metabolic activity and potentially in their relative abundance, in accordance with findings reported in the literature.^40^ The pH was stable throughout the whole experiment, with values of 7.5 ± 0.1 and 7.4 ± 0.1 for R-mag and R-ctrl, respectively (Figure 1d).

The total VFA (TVFA) present in the inoculum was initially rapidly consumed, with a subsequent gradual depletion observed during phase P1 (Figure 1b). In P2–P3, immediately after the shocks, an initial rise in TVFA concentration was noticed, in particular regarding the acetate (Figure 1, heatmap). This result can be attributed to a primary shock to the methanogens, who lately adapted to the new condition, as supported by the decreased TVFA of P3. Moreover, despite the identical conditions in which the two reactors were running, both propionate and acetate appeared to accumulate more in R-ctrl compared to R-mag (*p* = 0.02, Figure 1, heatmap). These variations may arise due to improper mixing during the first inoculum addition or stochastic events, leading to a different evolution of the microbial communities inside the two reactors. It is important to highlight that, while CH_4_ yield was decreasing throughout P2 and P3, TVFA were not accumulating, except in the initial periods of both phases. This evidence can be associated with the improved metabolism of SRB that was favored by the presence of SO_4_^2–^ and was consuming TVFA.^12,41^

### 3.2 Magnetite Addition Allowed the Recovery of Methane Yield and Resulted in H_2_S Removal

Magnetite supplement in R-mag during P4 led to a significant recovery (10 ± 1%, *p* = 0.049) in the methane yield (332 ± 19 mLCH_4_/gVS) compared to P3, and it was significantly higher (*p* = 1.6 × 10^–11^) compared to R-ctrl (277 ± 16 mLCH_4_/gVS) throughout the period (Figure 1a). Once magnetite was added in R-mag, the average CH_4_ content increased to 65 ± 1%, reaching 75% as the maximum level. This result is well in agreement with previous studies demonstrating methane production recovery after the supplementation of conductive materials to sulfate-loaded reactors.^42−44^ In parallel, the methane yield was also increased in R-ctrl; however, the average along P4 (295 ± 16 mLCH_4_/gVS) was 6% lower (*p* = 5.6 × 10^–6^) than the average value of the previous phase (314 ± 34 mLCH_4_/gVS). This evidence might be explained by a potential adaptation of methanogens to the increased SO_4_^2–^ concentration after a destabilization period that occurred during P3. During P4, the TVFA content sharply decreased by 90% in R-mag and by 86% in R-ctrl (Figure 1b). The drop might be associated with the manual feeding which was less precise compared to the calibrated pumps. Nevertheless, the TVFA had already shown a declining trend from day 97 until the end of P3, as it commonly happens in long-term operating reactors where the initial TVFA accumulated in the inoculum are consumed over time.^45,46^ In R-mag, TVFA decreased from 826 ± 6 to 161 ± 10 mg/L at the beginning and at the end of phase P4 (Figure 1b). Specifically, acetate and propionate showed a higher decrease (Figure 1b). In R-ctrl, TVFA dropped to a minor degree from the beginning and to the end of phase P4 (Figure 1b). The TVFA decrease was more pronounced in R-mag compared to that in R-ctrl (*p* = 0.00015). The results indicate that magnetite addition exacerbated the difference observed during the SO_4_^2–^ shock and enhanced the overall microbial metabolism, in particular, the acetogenic activity from propionate and butyrate, and the methanogenesis.^47^

H_2_S measurements after magnetite addition evidenced a massive decrease of H_2_S levels in R-mag that reached 16 ± 8 ppm in less than 7 days, while in R-ctrl the levels remained the same (5000 ± 200 ppm) compared to the previous phase. Considering that the microbiological insights could not support the changes in the H_2_S profile, the precipitation of H_2_S in the form of zerovalent sulfur or sulfur compounds due to the presence of magnetite was evaluated. Previous studies have demonstrated that the use of metal catalysts could convert H_2_S from gas streams to sulfur (S) at room temperature.^48−50^

An independent test was performed to assess wet catalytic oxidation (Table 1). The expected value for H_2_S dissolution in water at temperatures between 30 and 40 °C and pressure equal to 2.0 bar is reported to be between 2.0 and 3.0 gH_2_S/kgH_2_O.^51^ Given the parameters of the experiment, the expected dissolution should have reduced the overpressure from 1 to 0.5 bar. After 2 h from the H_2_S addition, pressure decreased both in the batch containing a S/Fe ratio equal to 1:9 and also in the one with a S/Fe ratio equal to 1:18 reaching values considerably lower compared to the control and calculated values (Table 1). The results demonstrated that H_2_S precipitated when magnetite was present in the batches.

**Table 1:** Overpressure Measurements to Assess the Dissolution and Precipitation of H_2_S in the Presence (Ratio S/Fe 1:9 and Ratio S/Fe 1:18) and Absence (Control) of Magnetite

	overpressure immediately after H_2_S addition (bar)	overpressure after 2 h from H_2_S addition (bar)	overpressure after 24 h from H_2_S addition (bar)
control	0.659 ± 0.012	0.569 ± 0.012	0.558 ± 0.008
ratio S/Fe 1:9	0.633 ± 0.013	0.135 ± 0.006	0.130 ± 0.007
ratio S/Fe 1:18	0.619 ± 0.007	0.133 ± 0.007	0.126 ± 0.008

**Figure 2:**
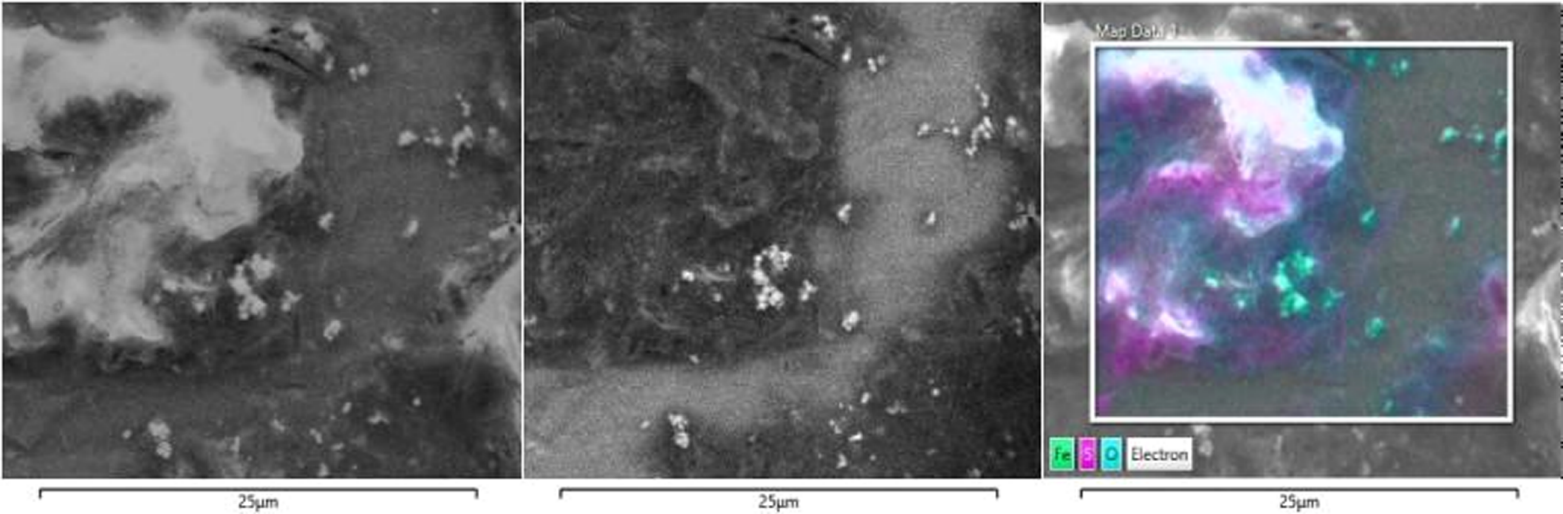
Scanning electron microscopy (SEM) of the magnetite particles. Analysis was performed to evaluate the presence of precipitated sulfur. From left to right: secondary electron detector (SED), backscattered electron detector (BSED), and scanning electron microscopy–energy-dispersive X-ray spectroscopy (SEM-EDX) images. The EDX analysis highlights the presence of sulfur (highlighted with pink color) precipitated with the magnetite (Iron—Fe, highlighted with green color; oxygen—O, highlighted with light blue color).

To further confirm this hypothesis, EDX analysis was performed on the magnetite extracted from the batch bottle with a S/Fe ratio S/Fe 1:9. Sulfur was detected in the analyzed sample, mixed with the magnetite (Figure 2). It is worth mentioning that EDX analysis cannot discriminate among different sulfur compounds. Therefore, it was not possible to distinguish whether the detected sulfur was in the form of zerovalent sulfur or FeS_2_ complexes. The precipitation process requires O_2_ in order to reoxidize Fe(II) to Fe(III).^52^ In the CSTRs operating under anaerobic conditions, the continuous flow replaced reduced magnetite without requiring further oxidation. Moreover, reductive metabolic reactions requiring electrons (e.g., CH_4_ and H_2_S production) may have supported magnetite reoxidation, as hypothesized by previous research where ferrous iron was suggested as an alternative electron donor in redox reactions occurring through long-distance electron transport.^53^

### 3.3 Microbial Community Composition and Dynamics: Unraveling the Effect of Magnetite

A genome-centric approach applied to the shotgun reads of the six samples allowed the reconstruction of 145 metagenome-assembled genomes (MAGs), with an average read alignment of 80% across all samples. The MAGs recovered in the present study were compared with those previously reported in a comprehensive MAGs database where a range of samples have been analyzed using a binning approach (http://microbial-genomes.org/).^94^ After clustering the MAGs at 95% average nucleotide identity, only 70 out of 145 MAGs identified in the present study represent species already deposited in the “global AD database” confirming that more than half of the species presented here were entirely new. After filtration according to the MIMAG standards,^54^ the remaining 108 MAGs had an average reads alignment of 70% and were selected for further analysis, including relative abundance in the samples and variation in terms of fold change, after magnetite addition in R-mag. The taxonomic investigation allowed the assignment of 12 MAGs at the species level and 26 MAGs at the genus level (Data S2), confirming the presence of a high fraction of uncharacterized species.

#### 3.3.1 Influence of SO_4_^2–^ and Magnetite on the Most Abundant Taxa

**Figure 3:**
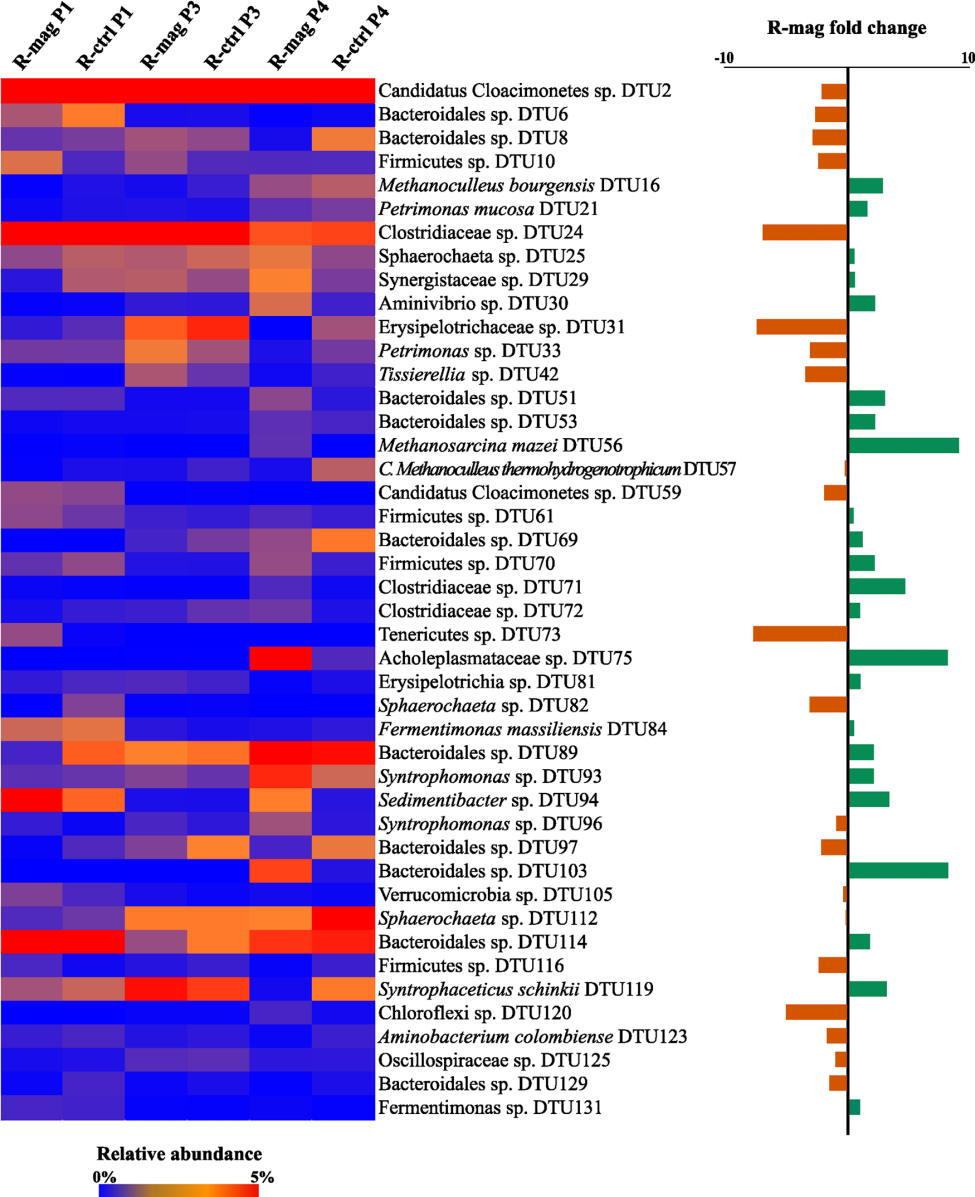
Heatmap representing MAGs with relative abundance higher than 0.5% in at least one period, with completeness >70%. Relative abundance of MAGs in the six samples (R-mag P1 and R-ctrl P1 are the samples taken before the SO_4_^2–^ shocks, R-mag P3 and R-ctrl P3 are the samples collected after the SO_4_^2–^ shocks, R-mag P4 and R-ctrl P4 are the samples collected after the magnetite addition in R-mag) is reported on the left part of the figure. Relative abundance values range from 0% (black) to 5% (red), and higher values are saturated (the color scale is in the top part). In the right part of the figure is reported the MAGs fold change in R-mag after magnetite addition (R-mag P4 vs R-mag P3); values lower than zero (orange) are on the left of the black line and values higher than zero (dark green) are on the right of the black line. Values range from −10 to 10. It must be considered that the metagenomic investigation method has limitations when it comes to providing precise absolute abundance values. As a result, the trends observed between sampling points may be influenced by the underestimated differences in the overall size of the microbial community. Phylogenetic information of the MAGs is reported in Figure S1, Supporting Data S1.

Taxonomic analysis showed that 19 and 50 of the 108 filtered MAGs were assigned to the Bacteroidetes and Firmicutes phyla, respectively (Data S3). Overall, the relative abundance of the phylum Firmicutes was not particularly affected under the different conditions. Nonetheless, when focusing on the family level, the presence of magnetite exerted a substantial influence on the relative abundance of Syntrophomonadaceae, since it increased from 2.3% in P3 to 6.7% in R-mag during P4, while it remained stable in R-ctrl throughout the entire experiment ranging from 1.9 to 2.7% (Data S3). This evidence suggested that members of the Syntrophomonadaceae family might be involved in DIET, as previously hypothesized,^55^ even though the mechanism is still to be clarified. The involvement of some Firmicutes species in polysaccharides degradation is a well-known property in many natural and engineered environments.^56,57^ The high relative abundance of Firmicutes can be attributed to their capability to degrade polysaccharides and oligosaccharides that constitute a considerable fraction of the biopulp.^56,57^ The plasticity of the AD microbiome is particularly evident in the top layers of the food chain (e.g., in the hydrolytic step), and this is determined by the high functional redundancy.^58,59^ According to this, it is highly possible that the species reported in the mentioned literature are different from those identified in the present study but still involved in the same functional process. The functional annotations showed the presence of genes involved in the carbohydrate metabolism (Data S2). In particular, Firmicutes DTU61 and Firmicutes sp. DTU70 had the gene encoding the β-fructfuranosidase (EC: 3.2.1.26), which catalyzes the formation of d-fructose and d-glucose 6-phosphate from sucrose 6-phosphate. In addition, Firmicutes sp. DTU61 and Firmicutes sp. DTU100 showed the presence of the 1,4-α-glucan branching enzyme (EC: 2.4.1.18) encoding the gene involved in the degradation of starch. This evidence confirms the involvement of Firmicutes spp. in the hydrolysis phase, specifically in carbohydrate degradation. Bacteroidetes were massively affected by the SO_4_^2–^ addition since their relative abundance decreased from 15 to 20 to 10% in both reactors (Data S3) and increased to 20% in phase P4 both in R-ctrl and in R-mag. These results suggest that Bacteroidetes adapted to the new condition and that magnetite addition seemed to have no specific effect on them (Figure 3). The observed trend is in line with previous studies which found that a lower TVFA concentration would favor the establishment of Bacteroidetes during mesophilic AD.^60,61^ Candidatus Cloacimonetes phylum, represented solely by two MAGs, was highly abundant in the initial inoculum, reached almost 20% of the microbial community after SO_4_^2–^ shock (Data S3), and then extensively declined during P4 in both reactors.

According to the literature, this phylum appeared to be active mainly during the initial step of cellulose hydrolysis or in the primary fermentation of hydrolysis products.^62^ Metagenomic analysis revealed the presence of two out of three genes of the propionyl-CoA metabolism (KEGG module M00741) in Candidatus Cloacimonetes sp. DTU2, suggesting its potential involvement in propionate oxidation. This evidence is supported by the literature, identifying the presence of the oxidative propionate degradation pathway in the Candidatus Cloacimonetes genome.^63^

Spirochaetes and Synergistetes phyla were represented by seven and six MAGs, respectively (Data S3). In R-ctrl, the H_2_S-rich environment promoted Spirochaetes growth, increasing in relative abundance from 3.7% in P1 to 8.1% in P4 (Data S3). The synergistic model between Spirochaetes and SRB was previously proposed, and Spirochaetes spp. were referred to as sulfur-oxidizing bacteria capable of detoxifying H_2_S-rich environments using oxygen or nitrate as electron acceptors.^64^ Nevertheless, the results showed that the removal of H_2_S in the reactors was complete and fast, indicating that Spirochaetes spp. alone could not remove the large amount of H_2_S present in the CSTRs. Nitrate can be present in traces in AD systems, and the low amounts could explain the slow process related to H_2_S oxidation using nitrate.^65^ These findings are in accordance with a previous study that highlighted the limited and slow removal of H_2_S by Spirochaetes spp.^64^ According to this, magnetite played a major role in the oxidation of H_2_S. In addition, members of the Spirochaetes phylum can degrade polysaccharides providing SRB with fermentation products such as acetate.^66^ Synergistetes increased in R-mag from 0.9 to 4%, while this trend was not observed in R-ctrl, where their relative abundance remained stable at around 2.5% during the entire process. Members of this taxon are known as syntrophic acetate-oxidizing bacteria and amino acid degraders.^67−69^ Both activities can provide substrates to methanogens, including, for example, H_2_, CO_2_, and acetate, suggesting that magnetite addition promoted the syntrophic behavior of Synergistetes.

#### 3.3.2 Metabolic Pathways Enriched under SO_4_^2–^ Shock and Magnetite Supplementation

**Figure 4:**
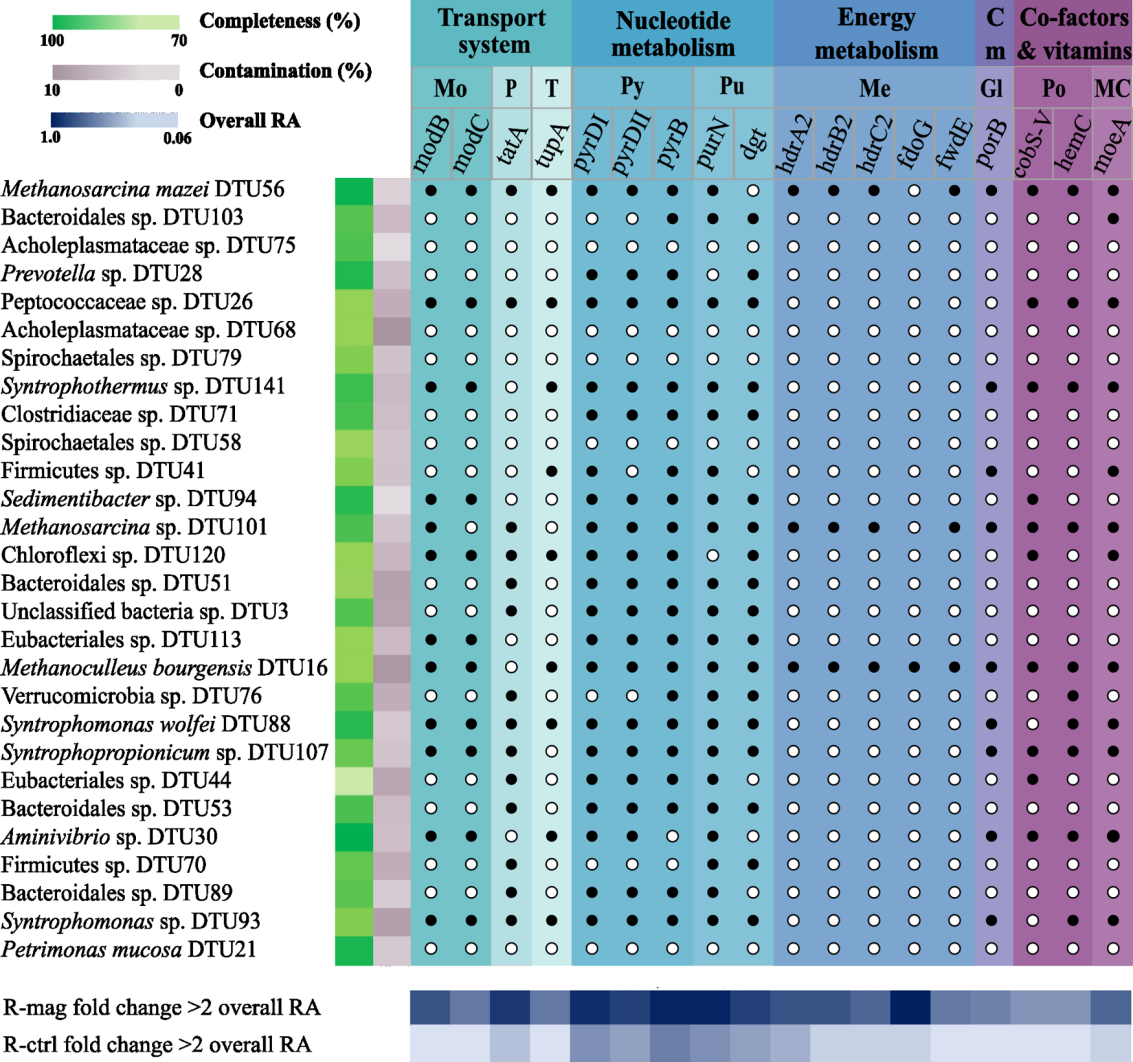
Graphical representation of the statistical analysis performed with enrichM. Two groups were compared: MAGs with fold change >2 in R-mag and in R-ctrl during P4. A subsection of the metabolic pathways enriched in R-mag is reported in the rows. Completeness and contamination are reported next to the assigned MAGs on the left part by the green and gray color scales, respectively. From top to bottom are reported: general metabolic pathway (C m is the acronym label for carbohydrate metabolism), label identifying the specific enriched module, and genes belonging to the module. Colored columns refer to the enriched modules. Presence/absence of genes is reported as black/white dots, respectively. Labels from left to right: molybdate transport system (Mo), sodium transport system (S), glutamate:Na^+^ symporter (G), sec-independent translocase for protein secretion (P), tungstate transport system (T), pyrimidine metabolism (Py), purine metabolism (Pu), methane metabolism (Me), pentose phosphate pathway (PPP), glycolysis (Gl), amino sugar and nucleotide sugar metabolism (ANS), porphyrin metabolism (Po), molybdenum cofactor biosynthesis (MC). At the bottom, a heatmap representing the overall relative abundance per each represented gene.

An unbiased analysis was performed to identify statistically enriched metabolic pathways in MAGs having higher relative abundance in selected samples (R-mag in P4 vs R-ctrl in P4). When comparing the MAGs enriched by more than two folds in R-mag (group 1) with those depleted (fold-change <2, group 2), a variety of metabolic pathways were found to be enriched (Data S4). In particular, the degradation and biosynthesis of carbohydrates and amino acids were more frequent in group 1 (*p*-values ranging from 0.02 to 0.04). A similar trend was found for genes involved in the metabolism of cofactors and vitamins (0.01 < *p* < 0.05, Data S4). These findings indicate that magnetite promoted the activity of taxa responsible for operating during hydrolysis and fermentation and, in general, improved the overall microbial community growth and metabolism. As a confirmation of what was observed during CSTRs monitoring, genes associated with methane production were enriched in group 1, underlining that magnetite enhanced archaeal growth (Data S4). Besides metabolic pathways, attention should be focused also on transport systems. The results highlighted that the twin-arginine translocation gene, *tatA*, was enriched in group 1 (*p* = 0.028), as well as different solute transport systems (*p* ranging from 0.01 to 0.04, Data S4). Prior research assessed that *tat* genes encode a protein transport system in many bacterial species involved in the biogenesis of bacterial electron transfer chains.^70^ Bacterial electron transfer chains interact directly with solute transport systems, coordinating energy production and solute transport.^71,72^ For instance, the respiratory chain proton gradient couples to other transport systems for nutrient uptake and ion transport. Furthermore, certain bacteria possess electron transfer chains that are coupled to the transport of metal ions or other specific substrates, enabling them to thrive in specific environments or under particular nutritional conditions.^71,72^ It can be hypothesized that magnetite stimulated the growth of Tat-encoding species, and this resulted in an enhancement of the solute transport systems, allowing the internalization of compounds that are further metabolized in catabolic and anabolic pathways. Nevertheless, it should be taken into account that this analysis is generated based on metagenomic data and a more comprehensive metabolic analysis should be carried out as further confirmation. When comparing MAGs with fold-change >2 in R-mag and MAGs with fold-change >2 in R-ctrl, the results showed that genes within the nucleotide, carbohydrate, methane, cofactors, and vitamins metabolisms (0.02 < *p* < 0.05, Data S5), as well as transport systems (0.01 < *p* < 0.05, Data S5) were enriched in the group belonging to R-mag, underlining once more the positive effect of magnetite on the AD process (Figure 4).

### 3.4 Microbial Dynamics of SRB and Methanogens: Syntrophism and Competition

The archaeal community was represented by five MAGs, of which two belonged to the *Methanosarcina* genus (*Methanosarcina mazei* DTU56, *Methanosarcina* sp. DTU101) and three belonged to the *Methanoculleus* genus (*Methanoculleus bourgensis* DTU16, *Candidatus Methanoculleusthermohydrogenotrophicum* DTU57, and *Methanoculleus* sp. DTU121). It is well documented in the literature that members of the genus *Methanosarcina* (e.g., *Methanosarcina barkeri*) can perform DIET by cooperating with syntrophic partners.^73^

H_2_S measurements after SO_4_^2–^ addition suggested a strong activity of SRB. Nonetheless, among all of the MAGs that presented at least one gene associated with sulfate reduction pathways, only one could be clearly defined as an SRB. Peptococcaceae sp. DTU26 had two genes out of three of the dissimilatory sulfate reduction pathway. This finding is in line with prior research identifying SRB within this taxon.^55,74^

Both methanogens and SRB can establish syntrophic interactions potentially based on DIET with syntrophic acetate-oxidizing bacteria (SAOB), syntrophic propionate-oxidizing bacteria (SPOB), and syntrophic butyrate-oxidizing bacteria (SBOB).^75^ Among the retrieved MAGs, 13 were identified as SBOB, eight were defined as SAOB, and only one was detected as SPOB (Data S2).

#### 3.4.1 Methanogens and SRB: From Cooperation to Competition

**Figure 5:**
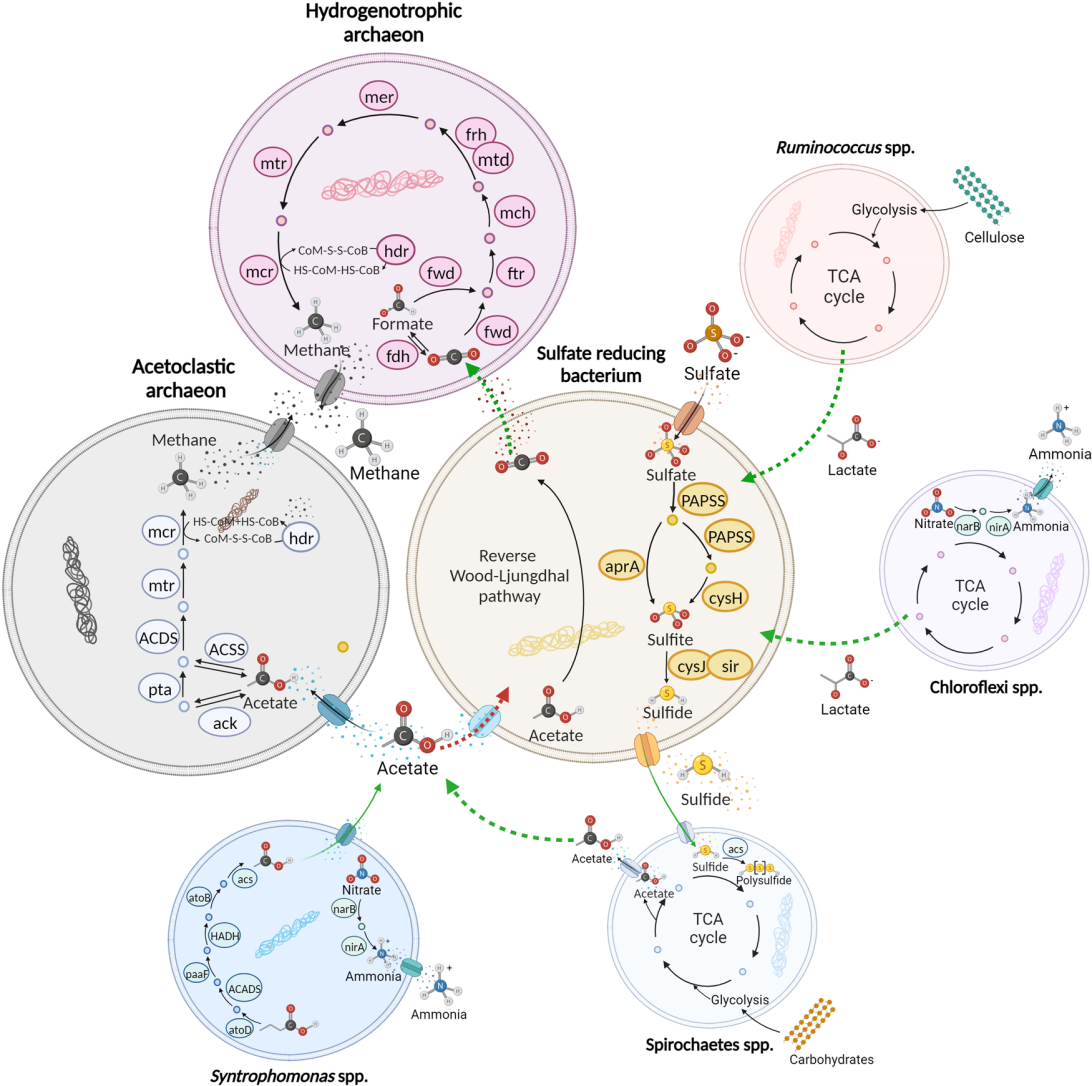
Potential interactions among syntrophic partners within the microbial community. Six representations, one per each taxon (Archaea, *Syntrophomonas*, Spirochaetes, *Ruminococcus*, and Chloroflexi) or group of microorganisms (e.g., SRB) are displayed to elucidate the interactions involving the main players, methanogens and SRB—(represented by bigger cells) with their potential syntrophic partners (represented by smaller cells). In each cell, the enzymes of a representative metabolism are drawn (Data S2): hydrogenotrophic methanogenesis in archaea, dissimilatory sulfate reduction and the reverse Wood–Ljungdhal pathway in SRB, and dissimilatory nitrate reduction in both Syntrophomonas and Chloroflexi. Substrates and products inside the cells are represented by small colored circles placed close to the arrows, while compounds exchanged among the cells are defined by their name, acronym, or chemical formula. Dashed green and red arrows indicate the main syntrophic and competitive interactions. Faded green lines represent possible direct compound exchanges between syntrophs. Created with BioRender.com, 2023.

Analysis of the biochemical and microbiological parameters suggested a potential competition between SRB and methanogens. Since both groups can consume acetate, H_2_, and CO_2_ for their metabolism, and sulfate reduction is thermodynamically favored compared to methanogenesis, SRB can outcompete methanogens.^2,5^ Magnetite addition could help electrotrophic archaea to thrive and restore their metabolic activity and relative abundance.^19,42^ The results indicated that both *Methanosarcina* spp. were clearly negatively affected by the addition of SO_4_^2–^ addition. After the second SO_4_^2–^ shock (P3), *M. mazei* DTU56 and *Methanosarcina* sp. DTU101 experienced a decrease in relative abundance ranging from 4- to 5.5-fold in both reactors. When magnetite was added in R-mag (P4), both increased in abundance with a fold change of 3 and 9, respectively, while they decreased by 5.5-fold in R-ctrl. Genes for cytochrome c biosynthesis (*ccmA-C, ccmE*, and *ccmF*, Data S2) were found in *M. mazei* DTU56, suggesting its potential involvement in DIET. On the contrary, *M. bourgensis* DTU16 and *C. Methanoculleusthermohydrogenotrophicum* DTU57 were favored by the presence of SO_4_^2–^, as confirmed by their relative abundance during P3 and P4. These findings revealed that SRB can have opposite interactions with acetoclastic and hydrogenotrophic methanogens (competitive and cooperative, respectively; Figure 5). A possible explanation is that SRB can use the Wood–Ljungdahl pathway in reverse to completely oxidize acetate to H_2_ and CO_2_,^76^ and they can also obtain electrons to produce hydrogen by increasing hydrogenase expression.^77^ The ability of SRB to produce H_2_ and CO_2_ might have helped methanogens that rely on hydrogenotrophic methanogenesis (e.g., *Methanoculleus* spp., Figure 5). Concurrently, acetate consumption by SRB may have hindered acetoclastic methanogens competing for the same substrate. Magnetite addition favored *Methanosarcina* spp. able to perform DIET, providing them the opportunity to compete more efficiently with SRB. Thereby, in the context of a competitive-cooperative relation between SRB and different methanogens, it is explained the observed fold change trend of *Methanosarcina* spp. and *Methanoculleus* spp. under the different conditions in P3 and P4.

With reference to potential SRB, both *Prevotella* sp. DTU28 and *Peptococcaceae* sp. DTU26 increased after magnetite addition in R-mag, with a fold change of 7.6 and 5.3, respectively, while in R-ctrl, only *Prevotella* sp. DTU28 increased with a fold change of 2.3. In addition, genes for cytochrome c biosynthesis (*ccmA, ccmE, and ccmF*) were identified in *Peptococcaceae* sp. DTU26. SRB participation in DIET has already been reported by a previous study, which evidenced the ability of SRB to directly accept electrons through the c-type cytochrome in the cell’s outer membrane for sulfate reduction (Table 2).^77^

**Table 2:** Table Containing the Copy Number Per Gene Stated in Figure 5 (Six Representative MAGs have been Chosen for the Representation while the Extended Analysis is Reported in Data S2)

	*Methanoculleus* sp. DTU121	*Methanosarcina* sp. DTU101	*Peptococcaceae* sp. DTU26	*Syntrophomonas* sp. DTU93	*Chloroflexi* sp. DTU130	*Spirochaetales* sp. DTU126	*Ruminococcus* sp. DTU23
ack		2				1	1
ACSS	3	1					1
pta		1				3	1
ACDS		1					
mtr	9	11					
mcr	4	5				2	
hdr	1						
fdh	1	1			3		
fwd	1	5					
ftr	1						
mch	1	2					
mtd	1	1					
frh	4	2					
mer	1	1					
cysJ							
sir							
cysH	1	3			2		1
aprA			1				
PAPSS			1		3		
atoD				1			
ACADS				7			
paaF				16			
HADH				6			
atoB				7			
acs							
nirA					1	3	
narB	3						
hydG							
TCAc					1/8	4/8	1/8

#### 3.4.2 DIET-Based Interactions between SRB/Methanogens and Syntrophic Partners

The experimental results showed that microbes known to establish syntrophic interactions with methanogens might also play a beneficial role toward SRB.^78−80^ Syntrophic acetate-oxidizing bacteria (SAOB, e.g., *Syntrophaceticus schinkii* DTU119 (Lee et al., 2016)) and syntrophic butyrate-oxidizing bacteria (SBOB—mainly represented by the genus *Syntrophomonas*)^81^ had an increased relative abundance in R-mag after magnetite addition (Data S2). While previous studies reported a rise in the relative abundance of SAOB and SBOB after magnetite addition, the mechanism behind the stimulatory effect is still unclear.^82,83^

In general, the presence of e-pili and cytochromes is often associated with the DIET mechanism.^8,10^ Indeed, the outcome of the metagenomic analysis evidenced that 12 MAGs, including, for example, *M. mazei* DTU56 and *Peptococcaceae sp. DTU26*, encoded the genes for cytochrome c biosynthesis, advising their potential involvement in DIET (Data S2). Magnetite can help the syntrophic interactions, establishing an electrical network that mediates long-range extracellular electron transfer.^84^ Moreover, many studies highlighted that magnetite could compensate for the lack of DIET-related microbial structures such as pilin and multiheme complexes—MHCs.^12,85−,87^ Under these circumstances, electron shuttles are required to transfer electrons between the inner and outer membranes.

Other MAGs seemed to be specifically associated with the presence of SRB. Particularly, *Ruminococcus* sp. DTU98, usually involved in cellulose fermentation to generate end products such as lactate and succinate, increased in both reactors after the SO_4_^2–^ shocks.^88,89^ This evidence suggested a potential syntrophic relationship between *Ruminococcus* sp. DTU98, producing lactate, and SRB, consuming lactate to produce H_2_S (Figure 5). In addition, MAGs assigned to the phylum Chloroflexi such as Chloroflexi sp. DTU120 and Chloroflexi sp. DTU130 were enriched after the SO_4_^2–^ shocks, and after the magnetite supplementation in R-mag, they increased by 3.1- and 1.5-fold, respectively. Moreover, Chloroflexi sp. DTU130 encodes genes for cytochrome c biosynthesis (*ccmA-C, ccmE*, and *ccmF*), suggesting its potential involvement as an electrogenic syntrophic partner in DIET, as previously reported.^90,91^ This interaction could be attributed to the fact that some genera belonging to the Chloroflexi phylum (e.g., *Anaerolinea*) produce lactic acid, which has been defined as a suitable electron donor for SRB (Figure 5).^92,93^ As previously discussed, members belonging to the Spirochaetes phylum seem to have a synergistic interaction with SRB. The results of the metagenomic analysis showed that Spirochaetales sp. DTU27 encoded one of the genes for the cytochrome c biosynthesis, suggesting that their syntrophic behavior might be based on DIET.

## 4 Conclusions

The current study investigated the effect of magnetite on AD of municipal biopulp. Magnetite addition resulted in an increased CH_4_ yield by 10%. Moreover, it allowed sulfur precipitation, reducing the H_2_S headspace content from ∼5000 to <20 ppm. Overall, magnetite supplementation benefited different microbial interactions with methanogens and SRB, involving diverse metabolic pathways and electron shuttles. As a consequence, AD DIET-based processes turn out to be an alternative strategy to promote the degradation of SO_4_^2–^-rich organic residues. As confirmed by the increased VFA consumption, magnetite addition constitutes a novel strategy to alleviate the impact of OLR fluctuations, which could significantly stress the methanogenic microbiome. Indeed, SRB, SAOB, and SBOB were found to be enriched when magnetite was added, highlighting their main role in promoting TVFA degradation. Finally, the relationships between SRB and archaea are found to be more complex than a mere competition for the same substrate; specifically, SRB can have either cooperative or competitive interactions with hydrogenotrophic and acetoclastic methanogens, respectively.

## Supporting Information Available

Characterization of inoculum and biopulp (Table S1) and Phylogenetic representation of the identified MAGs (Figure S1) (PDF)
Taxonomic and functional analyses performed on the MAGs identified using the genome-centric metagenomic approach (XLSX)
Taxonomic conversion from GTDBTk database to NCBI database and additional statistical analysis performed on the taxonomic groups (XLSX)
Results of the statistical analysis performed to identify enriched metabolisms in the MAGs with fold change >2 vs MAGs with fold change <2 in R-mag after magnetite addition (XLSX)
Results of the statistical analysis performed to identify enriched metabolisms in the MAGs with fold change >2 R-mag vs R-ctrl after magnetite addition vs R-ctrl after magnetite addition (XLSX)

## Notes

G.G.: Investigation, methodology, validation, data curation, formal analysis, writing—original draft. P.T.: Conceptualization, methodology, validation, data curation, writing—review editing. M.G.: Investigation, methodology. P.G.I.: Conceptualization, investigation. M.T.H.L.: SEM-EDX analysis. L.T.: Methodology, data curation, writing—review editing. P.K.: Supervision, methodology. S.C.: Conceptualization, supervision, methodology, data curation, writing—review editing. I.A.: Conceptualization, supervision, project administration, writing—review editing. All authors revised and have given approval to the final version of the manuscript.
