# Supplementary material for: Magnetite Alters the Metabolic Interaction between Methanogens and Sulfate-Reducing Bacteria
Source: Environ Sci Technol. 2023 Oct 20;57(43):16399–413. doi: 10.1021/acs.est.3c05948 (PMC10620991; doi:10.1021/acs.est.3c05948)
Supplement: Supplementary file 1 — es3c05948_si_001.pdf [file es3c05948_si_001.pdf]

Supplementary Table 1. Total solids (TS) and volatile solids (VS) characterization of inoculum and biopulp.

	TS (g)	AVG TS (%)	TS (g/L)	VS (g)	AVG VS (%)	VS (g/L)
Inoculum	0.30 ± 0.01	3.83 ± 0.17	38.31 ± 1.70	0.20 ± 0.02	67.75 ± 6.38	25.96 ± 3.27
Biopulp	0.87 ± 0.04	12.04 ± 0.21	120.43 ± 2.10	0.81 ± 0.03	92.80 ± 1.73	111.76 ± 1.16

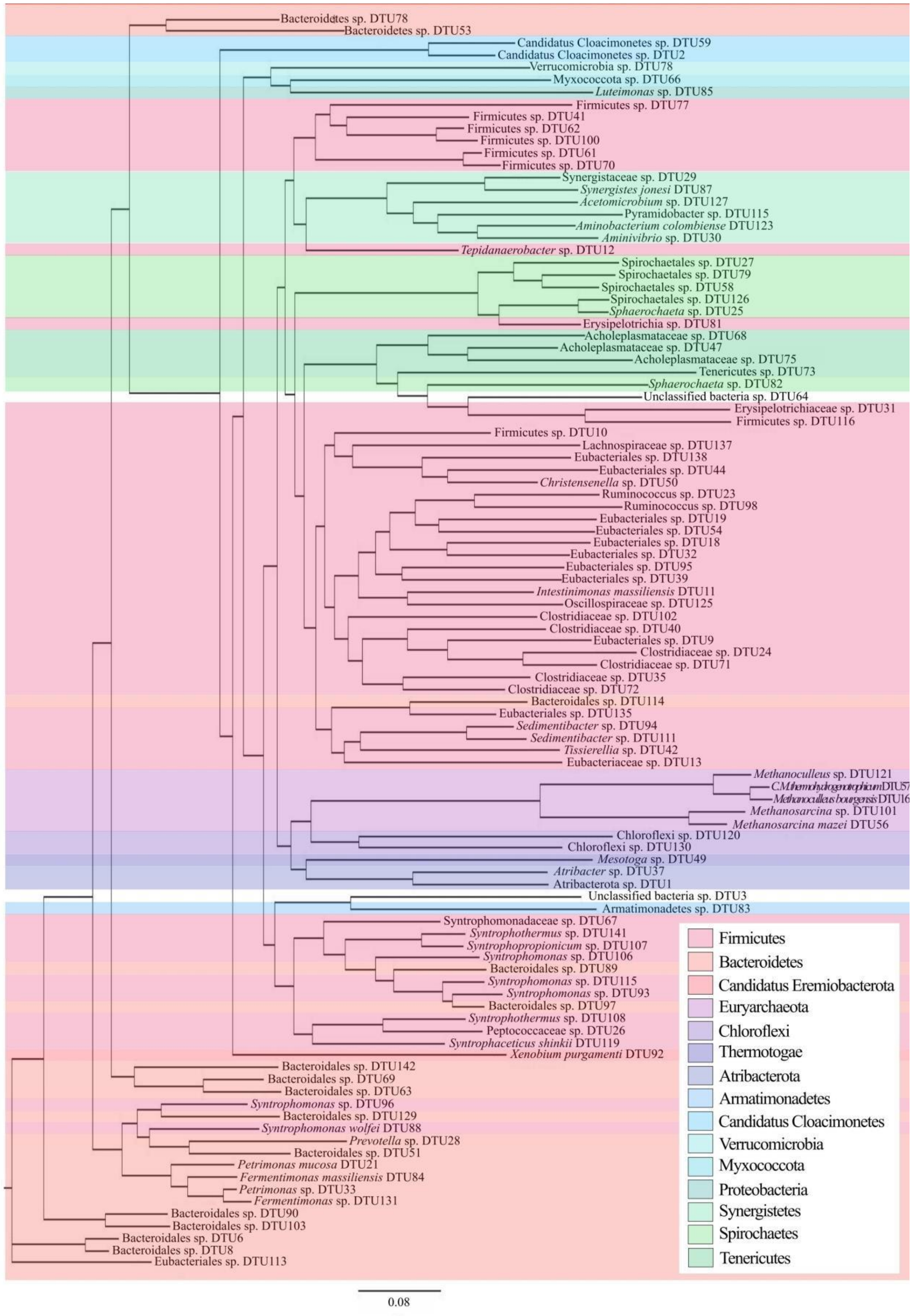

Figure S1. Phylogenetic representation of the identified MAGs. All the MAGs are reported in the phylogenetic tree. The colors represent the phyla identified. Some of the MAGs

belonging to the Firmicutes and the Bacteroidetes phyla are not correctly placed on the phylogenetic tree. The main explanation may be the contamination or limited genetic information available for these MAGs, making it difficult for the software to accurately determine their placement on the phylogenetic tree. Insufficient data can result in unresolved or misplacement of the MAGs. Moreover, the lack of reference data may lead to difficulties for the software to accurately place the MAGs on the tree. In addition, when MAGs represent mixtures of different organisms, incorrect phylogenetic placements can happen.
